# The Influence of Job and Individual Resources on Work Engagement Among Chinese Police Officers: A Moderated Mediation Model

**DOI:** 10.3389/fpsyg.2020.00497

**Published:** 2020-04-07

**Authors:** Ting Lan, Meirong Chen, Xiaoqing Zeng, Ting Liu

**Affiliations:** ^1^School of Psychology, Jiangxi Normal University, Nanchang, China; ^2^Department of Education, Nanchang Normal University, Nanchang, China

**Keywords:** job demand-resources model, work engagement, perceived organizational support, job satisfaction, regulatory emotional self-efficacy, Chinese police officers

## Abstract

**Background:**

The work engagement of police officers pertains to social stability and security, as well as to the orderly operation of the political-economic environment. Although there are many studies on work engagement at present, few studies focus on the influencing factors of police officers’ work engagement. According to the job demands-resources model and the conservation of resources theory, job resources (e.g., perceived organizational support) and personal resources (e.g., regulatory emotional self-efficacy) are important factors influencing work engagement. We assume that a moderated mediation model, in which job satisfaction plays a mediating role in the relationship between perceived organizational support and work engagement, regulatory emotional self-efficacy moderates not only the relationship between perceived organizational support and job satisfaction but also the relationship between job satisfaction and work engagement.

**Objective and Method:**

This study explores the drivers of work engagement through perceived organizational support and regulatory emotional self-efficacy among Chinese police officers using a convenient sampling method to administer a questionnaire to 744 Chinese police officers. A mediated model is proposed to investigate the mediating role of job satisfaction and the regulating role of regulatory emotional self-efficacy.

**Results:**

Job satisfaction mediated a positive relationship between organizational support and work engagement, and the perceived organizational support-job satisfaction and the job satisfaction-work engagement relationships were positively moderated by regulatory emotional self-efficacy, such that these relationships were stronger at higher levels of regulatory emotional self-efficacy. These findings have a practical significance for Chinese police officers’ work engagement advancement.

## Introduction

### The Definition and Significance of Work Engagement

With the rise in positive psychology research, work engagement, as a positive working state, has received extensive attention in the field of occupational health research. Kahn (1990) first defined work engagement as the way that “organization members control themselves to combine self and work roles.” Existing research generally defines work engagement as “a positive, fulfilling, work-related state of mind that is characterized by vigor, dedication, and absorption” (Schaufeli et al., 2002, p.74). Vigor refers to high levels of energy and mental resilience while working, the willingness to invest effort in one’s work, and persistence even in the face of difficulties. Dedication refers to strong employee involvement in their work with an experience of a sense of significance, enthusiasm, and challenge. Absorption refers to a state of employees being fully immersed and happily engrossed in their work, so that the experience of time spent working passes quickly. Studies have shown that work engagement not only predicts employee organizational commitment and proactive behavior (Bakker et al., 2012; Walden et al., 2017) but also helps in reducing burnout and other occupational stress symptoms, thereby improving individual mental health and well-being (Riipinen, 1997; Xanthopoulou et al., 2012). Therefore, research on work engagement is of great practical significance.

Compared to other professions, police work combines the unique features of stress, challenging tasks, high risk, and often unexpected events. In China, the phenomenon is even more pronounced. Specifically, policing is a risky service with a high casualty rate. The official website of The Ministry of Public Security of the People’s Republic of China announced that in 2019, 280 police officers and 147 auxiliary police officers died on duty, and 6211 police officers and 5699 auxiliary police officers were injured on duty (2019). Additionally, Chinese police have a heavy workload and strict work regulations. They often work under stressful conditions, and it is common for them to work overtime to deal with emergencies, which, in turn, increases psychological pressure and leads to physical and mental exhaustion (Liu et al., 2019). The work engagement of police officers is related to social stability and security, as well as to the orderly operation of the political-economic environment. Therefore, it is of practical significance to ensure that their work engagement is always at a high level.

The job demands-resources (JD-R) model (Bakker et al., 2003a, b, 2004; Bakker and Demerouti, 2007) has been used to predict work engagement (Bakker et al., 2007; Xanthopoulou et al., 2009). According to this model, any occupation contains risk factors associated with job stress, which fall broadly into two categories: job demands and job resources. Whereas job demands are the most important predictors of burnout, job resources are the most important predictors of work engagement (Schaufeli and Bakker, 2004; Halbesleben, 2010). Many studies have explored the antecedents of work engagement and maintained the traditional classification of these into two general categories: situational factors and individual factors (Maslach et al., 2001; Bakker and Demerouti, 2008). On the one hand, regarding situational factors, job resources have been identified as the main drivers of work engagement; job resources in turn leads to increased well-being and positive organizational outcomes (Bakker et al., 2014). Job resources mainly include physical, psychological, social, or organizational aspects of the job, and those that are found to predict work engagement include task variety, task significance, autonomy, feedback, social support from colleagues, a high-quality relationship with the supervisor, and transformational leadership. Job resources were correlated more strongly than job demands with engagement, and contributed to work engagement both daily and over time (Bakker et al., 2014). On the other hand, individual factors play an important role in work engagement. The empirical evidence suggests that both higher-order individual factors (i.e., emotional stability, extraversion, conscientiousness, proactive personality) and lower-order individual factors (also called personal resources, i.e., self-efficacy, optimism, and self-esteem) are positively related to work engagement (Bakker et al., 2014).

This paper uses the perspectives of both job resources and personal resources in the JD-R model to explain the work engagement of police officers in the context of Chinese culture.

### The Relationship of Perceived Organizational Support on Work Engagement

Regarding job resources, perceived organizational support as an important psychological resource has a significant impact on work engagement. Perceived organizational support refers to employees’ perception of the extent to which their organization values their contributions and is concerned about their happiness (Eisenberger et al., 1986). Studies have revealed a significant, positive correlation between organizational support and work engagement based on employees’ perspectives. Furthermore, organizational support affects their work engagement (Bakker and Demerouti, 2007; Kou, 2012; Murth, 2017), as well as their work attitudes and behaviors (Coyle-Shapiro and Conway, 2005). Consequently, organizational support (including supervisors and colleagues) has an important impact upon work engagement.

The conservation of resources (COR) theory (Hobfoll, 1989, 2002) states that people strive to acquire and protect resources that they find useful. In real life, people face various pressures. When the resources they have cannot withstand the challenges they face, pressures develop. In order to alleviate these pressures, individuals will always seek opportunities to obtain new resources to meet these needs or to avoid the consumption of resources. Additionally, when individuals obtain more resources, the possibility of losing existing resources is reduced. Individuals will also use these resources to obtain other resources, thus increasing the total existing amount of existing resources. That is, if the resources (e.g., support) obtained by police officers from the organization are not enough, police officers cannot effectively cope with the pressure they bear. In order to reduce the pressure brought on by their work, their work engagement will be correspondingly reduced to avoid the consumption of resources. When the police organization has abundant resources, police officers may obtain other resources and have the ability to cope with the pressure they face at work, so as to immerse themselves into the work with a positive state.

In summary, organizational support is an effective predictor of work engagement, but the potential reason underlying this relationship remains unclear. Determining the relationship between perceived organizational support and work engagement is of great significance in improving police officers’ active participation in their work. Testing moderated mediation models on police officers may help explain how perceived organizational support relates to work engagement. Moreover, according to the JD-R model, both job resources and personal resources are important drivers of work engagement. Further, the COR theory also argues for bridges between environmental and individual theories and provides explanations for dynamic processes. Therefore, job resources and personal resources are not isolated, but what is the relationship between them? How they interact on work engagement is worth further investigation. Therefore, from the perspective of organizational and personal resources, this study attempts to analyze the work engagement of police officers in the context of Chinese culture. Exploring the mediating and moderating variables underlying this association may advance scholars’ understanding of how and when perceived organizational support can be employed in order to promote police officers’ work engagement.

### Job Satisfaction as a Mediator

Job satisfaction represents “the positive emotional reactions and attitudes an individual has toward their job” (Faragher et al., 2005, p. 106). Although studies have shown that job satisfaction is functionally similar to work engagement (e.g., both structures reflect positive motivational states that drive performance), the two are not synonymous. According to the circumplex model of affect proposed by Russell and Carroll (1999), researchers (Guglielmi et al., 2016) distinguish the affective states as a linear combination of two dimensions: activation/arousal and pleasure. Job satisfaction therefore represents an affective state reflecting a high level of pleasure and a low level of arousal, while, on the contrary, work engagement is characterized by high levels of both pleasure and activation.

Job satisfaction is a function not only of the objective properties of a job but also of “the pleasurable emotional state resulting from the appraisal of one’s job as achieving or facilitating one’s job values” (Locke, 1969). As the result of evaluating the ultimate value of work, job satisfaction can be seen as a scale for measuring the significance of one’s own work. Against the background of Chinese culture, police officers, who practice a profession with high social status, have a high sense of self-identity invested in their profession and consider the social significance of their profession very high (Liu et al., 2019). When the external resources of police officers (such as the support provided by the organization) are sufficient, their socio-emotional needs are met, and they are likely to report more positive job attitudes, including job satisfaction. At the same time, job satisfaction, as an individual’s subjective affective state, has a pleasant emotional response due to the realization of the individual’s work value, and the individual may then be more actively engaged in the work and more strongly committed to their tasks. Rhoades and Eisenberger (2002) conducted a meta-analysis of over 70 studies on organizational support, which they found can increase employee job satisfaction and positive behavior at work, thereby encouraging employee engagement. Therefore, we take job satisfaction as a mediating variable and combine the external and the self to explore the methods and basis of police officers’ work engagement in greater detail.

On one hand, a strong perception of organizational support can promote job satisfaction. Previous studies have shown a close relationship between employee organizational support and job satisfaction (Halbesleben, 2010; Miao, 2011; Hashish, 2015). The COR theory states that those who lack resources are not only more vulnerable to resource loss but also that the initial loss begets future loss. In contrast, those who possess resources are more capable of gain, and initial resource gain begets further gain. Thus, organizational support as an external resource provides the external energy that individuals use to gain a sense of hope at work and can satisfy the resources that they consume while dealing with work stress. Because job resources include potential motivation, constantly motivating employees to pursue their goals results in higher performance and job satisfaction. Prior studies have shown that perceived organizational support plays both an important role in determining job satisfaction and is a significant predictor of job satisfaction (Eisenberger et al., 1997; Kwak et al., 2010). Thus, perceived organizational support has a significant impact on job satisfaction.

On the other hand, individuals with higher job satisfaction are more likely to be engaged in work. Previous research results found that job satisfaction is positively associated with work engagement (Brough and Biggs, 2015; Mudrak et al., 2017). That is, when individuals’ socio-emotional needs are met and they are satisfied with their work, they devote themselves to that work in a more active and fuller state of mind and are able to realize their work value. However, if individual job satisfaction is low, their work engagement decreases (Ćulibrk et al., 2018). Therefore, individuals’ job satisfaction may be related to their work engagement.

As an important factor of work engagement, perceived organizational support may be related to individual work engagement through individual job satisfaction. Therefore, we propose the following:

Hypothesis 1: Job satisfaction mediates the relationship between organizational support and work engagement among Chinese police officers.

### Regulatory Emotional Self-Efficacy as a Moderator

Work engagement is influenced not only by contextual factors such as perceived organizational support but also by individuals’ internal characteristics (Brown, 1996). Within the framework of COR theory, two major resource types—individuals (e.g., self-efficacy) and psychosocial resources— are examined (Hobfoll et al., 2003). Studies have demonstrated that self-efficacy controls the relationship between perceived organizational support and job participation to a certain extent (Caesens et al., 2014). As a personal resource, self-efficacy can be considered an important resource that people want to protect. Perceived self-efficacy (Bandura, 1997,2006) concerns people’s beliefs regarding their ability to influence events, which forms the basis of human motivation and also plays a crucial role in the self-regulation of emotional states. Regulatory emotional self-efficacy concerns an individual’s confidence in his or her ability to handle his or her emotional state (Bandura et al., 2003), which plays an important role in managing individual behavior and personality. Caprara et al. (2006) highlighted the wide variation in people’s individual differences in managing their emotional experiences in daily life on account of not only their diverse abilities to manage their emotions but also of their unique beliefs in terms of emotional self-regulation. Research shows that a short-term positive emotional experience can build lasting psychological resources and trigger a spiral of emotional well-being (Fredrickson and Joiner, 2002). In addition, research has shown that, measuring emotional regulation self-efficacy can effectively predict and regulate emotional state and related areas (Zeng et al., 2019). Therefore, regulatory emotional self-efficacy, as a personal resource of emotional regulation, is highly important when an individual deals with negative emotions in the face of inadequate or limited organizational support.

The job characteristics model (Hackman and Oldham,1976,1980) examines individual responses to jobs (e.g., job satisfaction) as a function of job characteristics moderated by individual characteristics (Roberts and Glick, 1981). According to the JD-R model, when job resources (e.g., organizational support) are insufficient, police officers can invoke individual resources (e.g., regulatory emotional self-efficacy). Police officers who have a high sense of regulatory emotional self-efficacy are more able to deal with or face negative emotions at work and take a more positive view of their work. They can also deal with the negative emotions caused by stress and risk events more effectively. As a result, police officers with higher regulatory emotional self-efficacy may have higher job satisfaction than those with lower regulatory emotional self-efficacy.

In addition, as mentioned above, job satisfaction and work engagement are two different concepts. When job satisfaction is low, police officers with a high level of regulatory emotional self-efficacy can take corresponding measures to adjust their emotions, so that they can devote themselves to their meaningful work in a better emotional state (i.e., their work engagement is also high). Officers with a low level of regulatory emotional self-efficacy may not be able to deal with negative emotions effectively, and, therefore, cannot engage in work in a positive state. Therefore, we propose the following:

Hypothesis 2(a): Compared with the police officers with low regulatory emotional self-efficacy, when the police officers’ regulatory emotional self-efficacy is high, the perceived organizational support would play a greater role in promoting job satisfaction. Hypothesis 2(b): Similarly, compared with the police officers with low regulatory emotional self-efficacy, when the police officers’ regulatory emotional self-efficacy is high, job satisfaction would also play a greater role in promoting work engagement.

Hypothesis 3: The mediating role that job satisfaction would play in the relationship between perceived organizational support work engagement among Chinese police officers would be moderated by regulatory emotional self-efficacy.

The JD-R model indicates that both job resources and personal resources have important influence on work engagement. The COR theory holds that resources do not exist in isolation. Thus, resources such as perceived organizational support, job satisfaction, and regulatory emotional self-efficacy are important in their own right and have their own influence on work engagement. These influences are seldomly singular; rather, they appear in groups such that they attract and form building blocks with each other. However, the interaction between organizational factors and personal factors and that interaction’s internal relationship remain unclear. Based on the JD-R model and COR theory, mediation and adjustment analyses can be used to determine the relationships between these variables, thus making it a worthy topic for further study. In addition, existing research has mainly focused on police officers in Western nations, particularly the United States (Lambert et al., 2015). Thus, it is necessary to perform research involving police officers from an array of countries, including non-Western nations. Furthermore, under the condition that the organizational support is established, if a current work assignment must be completed within a deadline, individuals may need to increase work engagement by evoking the role of personal factors by, for example, improving the ability of emotional regulation self-efficacy, increasing job satisfaction, and so on. In other words, when the situation (organizational support) cannot be changed, the police officers can adjust their emotional state to ensure the effective task completion. Therefore, the main purpose of this study is to investigate the relationship among organizational support, job satisfaction, emotional regulation, self-efficacy, and work engagement.

**FIGURE 1:**
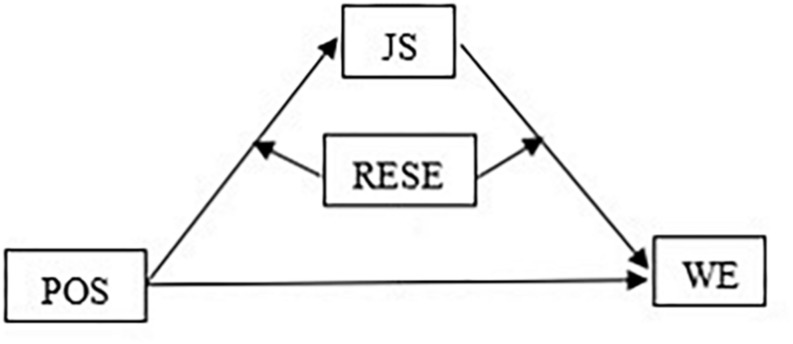
Hypothesis model. POS, perceived organizational support; JS, job satisfaction; WE, work engagement; RESE, regulatory emotional self-efficacy.

Considering these issues together, we constructed a moderated mediation model (see Figure 1) to provide a full understanding of the relationship between perceived organizational support and Chinese police officers’ work engagement. We assume that job satisfaction plays a mediating role in the relationship between perceived organizational support and work engagement, and that regulatory emotional self-efficacy moderates not only the relationship between perceived organizational support and job satisfaction but also the relationship between job satisfaction and work engagement.

## Methodology

### Participants

We administered a survey to 800 police officers from China in the provinces of Nanchang and Jiangxi during the period of centralized training and learning in July 2018. We conducted a field pen-and-paper test. Two documents were given to the police, including an informed consent form, which stated that the survey was conducted anonymously, that the results were for academic research only, and that all information will be kept confidential. All 800 participants signed the informed consent form before answering the questionnaire. The other document was a questionnaire comprising the Perceived Organizational Support Scale, Job Satisfaction Survey, Regulatory Emotional Self-Efficacy Scale, and Utrecht Work Engagement Scale. We distributed 800 questionnaires; during the process of manual data input from paper-and-pencil questionnaires, questionnaires with partially missing data (i.e., partially complete surveys), potentially from random responding, were excluded, leaving 744 valid questionnaires, with an effective recovery rate of 93%.

The participants included 609 males and 135 females. The average age was 35.45 (*SD* = 8.13) and the mean seniority was 9.88 years (*SD* = 10.15). Among police classifications, 252 officers handle crime (33.87%), 226 work in traffic enforcement (30.38%), and 266 fall into other categories (35.75%). In terms of education level, 106 had a junior college diploma (14.25%), 597 had bachelor’s degrees (80.24%), and 41 had master’s degrees (5.51%). The Ethics Committee of the College of Psychology at Jiangxi Normal University approved this study.

### Measurements

#### Perceived Organizational Support Scale

Eisenberger et al. (1986) developed the organizational support scale, which is a simplified version comprising the most important nine items from the original scale (two items are scored in reverse). An example item is, “My organization really cares about my well-being.” The reduced scale has good reliability, above 0.90 (Wayne et al., 2002; Chen et al., 2005). It is scored on a 7-point Likert scale, from 1 (strongly disagree) to 7 (strongly agree), with the higher score indicating a greater sense of organizational support. In this study, the scale had good internal consistency (Cronbach’s α = 0.91).

#### Job Satisfaction Survey

We used a job satisfaction survey from Minnesota (Maslach and Jackson, 1981) with 20 items. Example items are “The chance to be ‘somebody’ in the community” and “The chance to try my own methods of doing the job.” The participants rated each item on a 5-point Likert scale ranging from 1 (very dissatisfied) to 5 (very satisfied). These 20 items mainly measure internal satisfaction (e.g., personal value, learning opportunities, development space), and external satisfaction (e.g., remuneration, promotion opportunities and management), but since we are interested in the overall structure, we aggregated the scores of all sub-scales to obtain a total job satisfaction score. A higher score indicated greater job satisfaction. In this study, the scale had good internal consistency (Cronbach’s α = 0.93).

#### Regulatory Emotional Self-Efficacy Scale

We used the Chinese version of the Regulatory Emotional Self-Efficacy Scale developed by Caprara et al. (2008). It was created to assess perceived self-efficacy in managing negative (NEG) emotions and in expressing positive (POS) affect. NEG was represented by despondency-distress (DES) and anger-irritation (ANG). Research shows that for the Chinese samples, regulatory emotional self-efficacy is divided into three dimensions: POS, DES, and ANG (Wen et al., 2009). Example items include “Express joy when good things happen to you?” (POS), “Keep from getting dejected when you are lonely?” (NEG), and “Manage negative feelings when reprimanded by your parents or significant others?” (ANG). We provided a 5-point Likert scale for the participants to rate the items from 1 (totally inconsistent) to 5 (completely consistent), with a higher score signaling better self-efficacy of emotion regulation. In this study, the scale had good internal consistency (Cronbach’s α = 0.90).

#### Utrecht Work Engagement Scale

We employed the Chinese version of the 15-item Utrecht Work Engagement Scale (UWES), developed by Schaufeli et al. (2002) and translated by Zhang and Gan (2005). In the Chinese version of UWES, two items (one item for the “dedication” and “absorption” subscales) with an unsatisfactory discrimination index were deleted to determine the official version. It includes three subscales: (1) “vigor,” (2) “dedication,” and (3) “absorption.” An example item for each resource includes: “At my work, I feel full of energy” (vigor), “I am proud of the work that I do” (dedication), and “When I am working, I forget everything else around me” (absorption). All items were rated on a 7-point Likert scale ranging from 0 (never) to 6 (always), with higher scores implying better work engagement. In this study, the scale had good internal consistency (Cronbach’s α = 0.91).

### Data Analysis

We applied the prior procedural control process of test and common variance analysis to the four questionnaires using the Harman’s single-factor test (Podsakoff and Mackenzie, 2003) and one factor model test. Using SPSS 22.0 statistical software, we first carried out the difference test for each variable, and then used Pearson’s correlation to determine the correlations between the variables. We used gender, police category, and educational level as control variables in the subsequent analysis in order to exclude their influence. Using AMOS24.0, we carried out a confirmatory factor analysis to test the measurement model.

We tested the mediating effect of job satisfaction and the moderating effect of emotional regulation of self-efficacy using a moderated mediation analysis. We applied the percentile bootstrap method based on deviation correction and used the SPSS macro program PROCESS v3.0 (written by F. Andrew and edited by Hayes, 2013). By extracting 5,000 bootstrap samples (each with a sample size of 744), we obtained a robust standard error and bootstrap confidence interval (CI) of the parameter estimation. We tested the mediating effect of job satisfaction on the relationship between perceived organizational support and work engagement (Model 4: mediation model), and the whole moderated mediation hypothesis (Model 59: moderated mediation model). In order to determine how regulatory emotional self-efficacy impacts perceived organizational support, job satisfaction, and work engagement, we carried out a simple slope test. We also utilized the interaction diagram based on regulatory emotional self-efficacy (one standard deviation above the mean and one standard deviation below it).

## Results

### Assessment of Common Method Bias and Confirmatory Factor Analysis

Common Method Variance (CMV) is caused by the artificial co-variation between independent variables and dependent variables caused by the same measurement environment, project context or project characteristics of the same data source or grader. Such artificial co-variation between latent variables will mislead the research results and conclusions.

Therefore, in this study, we randomly split the sample into two halves. In half of the samples, we performed exploratory factor analysis (EFA) without rotation for all variable items. The results showed eight factors (e.g., vigor, dedication, absorption) with a characteristic root greater than 1; the variance explained by the first factor was 34.41%, which is less than 40% of the critical standard (Podsakoff and Mackenzie, 2003; Hayes, 2018). In the other half samples, we performed a confirmatory factor analysis (CFA), and the one factor model fitting indexes were χ^2^ = 1563.42, *df* = 170, χ^2^/*df* = 9.20, RMSEA = 0.15, IFI = 0.62, CFI = 0.62, and TLI = 0.53, all of which were lower than the judging criteria. Which indicates that the common method bias has little influence on the results of this study. In addition, we used the control for effects of an unmeasured latent methods factor to test whether there was a common method bias (Podsakoff et al., 2012). The results show that the improvement degree of the model’s fitting index is not high after adding the common method factor into the four factors, as the increase range of CFI and TLI is less than 0.01, and the decrease range of RMSEA is less than 0.02 (as shown in Table 1), indicating that the fitting data of the model with method factor is not significantly improved, indicating that although the common method bias may exist, it has less impact on this study. Therefore, there was no serious common method variance in this study.

**TABLE 1:** Confirmation factor analysis.

Fitting index	χ^2^	*df*	χ^2^/*df*	RMSEA	CFI	TLI	IFI
One factor model	2544.26	170	14.97	0.14	0.65	0.61	0.65
Two factors model	2034.77	169	12.04	0.13	0.72	0.69	0.72
Three factors model	1891,07	167	11.32	0.12	0.74	0.71	0.75
Four factors model	435.86	164	2.66	0.05	0.96	0.95	0.96
Four factors + Method factor	294.14	146	2.02	0.05	0.94	0.94	0.92

*Four factors model (WE, POS, JS, RESE); Three factors model (WE + POS, JS, RESE); Two factors model (WE + POS + JS, RESE); One factors model (WE + POS + JS + RESE). *N* = 744; WE, work engagement; POS, perceived organizational support; JS, job satisfaction; RESE, regulatory emotional self-efficacy. RMSEA, root mean square error of approximation. CFI, comparative fit index. TLI, Tucker-Lewis index. IFI, incremental fit index.*

Before testing the mediation effect, the confirmatory factor analysis was used to test the measurement model. There are four latent variables used in this study, namely, work engagement, perceived organizational support, job satisfaction, and regulatory emotional self-efficacy. As can be seen from Table 1, χ^2^ = 435.86, *df* = 164, χ^2^/*df* = 2.66, RMSEA = 0.05, CFI = 0.96, TLI = 0.95, IFI = 0.96. All the fitting indexes reached the criteria, indicating that the four factors model fitted well, and the fitting coefficients were better than those in the three factors model, the two factors model, and the one factor model. It indicates that the variables have good discriminant validity.

### Descriptive Statistics and Correlation Analysis

The outcomes of the difference test revealed significant gender differences in job satisfaction and emotional self-regulation (*t* = −2.12, *p* < 0.05, *d* = 0.44; *t* = −3.29, *p* < 0.01, *d* = 0.34). The degrees of perceived organizational support, regulatory emotional self-efficacy, job satisfaction, and work engagement all varied significantly by police classification and education level. Therefore, we used gender, police classification, and cultural level as control variables to exclude their influences in the subsequent analysis. We carried out a correlation analysis on police officers’ perceived organization support, regulatory emotional self-efficacy, job satisfaction, and work engagement. The findings illustrated a significant, positive correlation between the variables. The magnitude and direction of the correlation coefficient were in line with the expectations (as shown in Table 2).

**TABLE 2:** Means, standard deviations, and correlation matrix of all variables.

	*M*	*SD*	1	2	3	4
(1) WE	53.75	14.74	1			
(2) POS	45.39	10.55	0.38***	1		
(3) JS	72.22	11.68	0.65***	0.60***	1	
(4) RESE	44.45	7.71	0.36***	0.46***	0.46***	1

*N* = 744; WE, work engagement; POS, perceived organizational support; JS, job satisfaction; RESE, regulatory emotional self-efficacy. ***P < 0.001.

### The Relationship Between Perceived Organization Support and Work Engagement: Moderated Mediation Test

Correlation analysis revealed that the relationship between perceived organization support, regulatory emotional self-efficacy, job satisfaction, and work engagement met the conditions for the mediating effect test.

**FIGURE 2:**
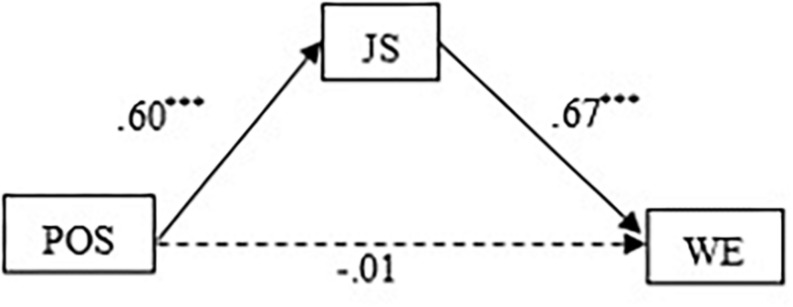
Path coefficients of perceived organizational support, job satisfaction and work engagement. *N* = 744, POS, perceived organizational support; JS, job satisfaction; WE, work engagement. ***P < 0.001.

First, we used the PROCESS macro for SPSS (Model 4) to test Hypothesis 1. Table 3 indicates that perceived organizational support significantly and positively predicted job satisfaction (β = 0.38, *t* = 12.91, *p* < 0.001), but when we put both organizational support and job satisfaction into the regression equation, the direct prediction effect of organizational support on work engagement was not significant (β = 0.04, *t* = 0.58, *p* > 0.05). However, perceived organizational support can indirectly and significantly predict work engagement through job satisfaction (β = 0.67, *t* = 17.95, *p* < 0.001). Furthermore, the 95% bootstrap CI of the mediating association did not contain zero ([0.59,0.74]), the proportion of the mediating effect in the total effect (ab/c) was 63.5%. Altogether, the results support Hypothesis 1. Figure 2 displays the specific path coefficients among the variables.

**TABLE 3:** Regression analysis results of the mediating role of job satisfaction between perceived organizational support and work engagement.

Regression equation		Overall model fit			Regression coefficient significance			
Outcome	Predictor	*R*	*R*^2^	*F*	β	LLCI	ULCI	*t*
JS	Gender	0.66	0.43	128.47***	0.19	0.01	0.05	2.74**
	Police classification				−0.06	−0.09	−0.03	−4.18***
	Education level				0.02	−0.07	0.11	0.38
	POS				0.60	0.54	0.66	19.53***
WE	Gender	0.69	0.48	124.31***	−0.02	−0.15	0.12	−0.23
	Police classification				−0.04	−0.07	−0.01	−2.90**
	Education level				0.11	0.02	0.20	2.47*
	POS				0.04	−0.01	0.09	0.58
	JS				0.67	0.59	0.74	17.95***

*N* = 744; WE, work engagement; POS, perceived organizational support; JS, job satisfaction. All the variables in the model were normalized and then substituted into the regression equation. LLCI, lower level confidence interval. ULCI, upper level confidence interval. *P < 0.05, **P < 0.01, ***P < 0.001.

**FIGURE 3:**
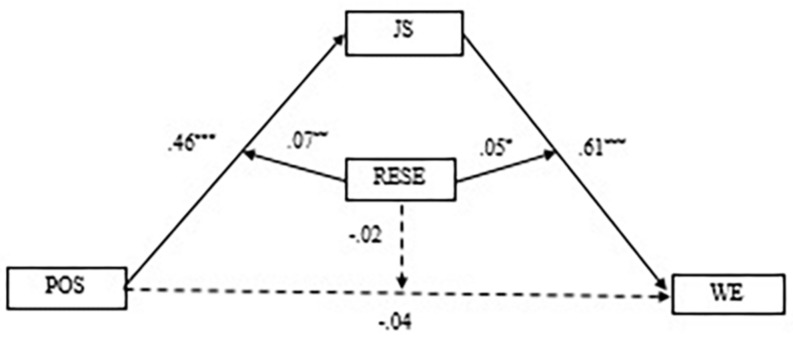
Path coefficients of perceived organizational support, job satisfaction, regulatory emotional self-efficacy and work engagement. *N* = 744, POS, perceived organizational support; JS, job satisfaction; WE, work engagement; RESE, regulatory emotional self-efficacy. *P < 0.05, **P < 0.01, ***P < 0.001.

Next, we employed the PROCESS macro for SPSS (Model 59) to test the whole moderated mediation hypothesis. The results showed (see Table 4), in the direct path, perceived organizational support did not significantly predict work engagement (β = -0.04, *t* = −1.07, *p* > 0.05), regulatory emotional self-efficacy significantly and positively predicted work engagement (β = 0.15, *t* = 4.50, *p* < 0.001), and the interaction items of perceived organizational support and regulatory emotional self-efficacy had no significant predictive effect on work engagement (β = −0.02, *t* = −0.75, *p* > 0.05). This suggests that regulatory emotional self-efficacy did not moderate the direct path. In the first half of the path, perceived organizational support significantly and positively predicted job satisfaction (β = 0.46, *t* = 15.03, *p* < 0.001), regulatory emotional self-efficacy significantly predicted job satisfaction (β = 0.33, *t* = 11.11, *p* < 0.001), and the interaction items of perceived organizational support and regulatory emotional self-efficacy had a significant and predictive effect on job satisfaction (β = 0.07, *t* = 3.00, *p* < 0.01). This implies that regulatory emotional self-efficacy plays a moderating role in the relationship between perceived organizational support and job satisfaction. In the latter half of the path, job satisfaction significantly and positively predicted work engagement (β = 0.61, *t* = 15.30, *p* < 0.001), regulatory emotional self-efficacy significantly and positively predicted work engagement (β = 0.15, *t* = 4.50, *p* < 0.001), and the interaction items of job satisfaction and regulatory emotional self-efficacy had a significant predictive effect on work engagement (β = 0.05, *t* = 2.15, *p* < 0.05). This indicates that regulatory emotional self-efficacy plays a moderating role in the relationship between job satisfaction and work engagement. Figure 3 displays the specific path coefficients among the variables.

**TABLE 4:** Regression analysis results of regulatory emotional self-efficacy moderate the mediation process.

Regression equation		Overall model fit			Regression coefficient significance			
Outcome	Predictor	*R*	*R*^2^	*F*	β	LLCI	ULCI	*t*
JS	Gender	0.72	0.52	122.15***	0.15	0.03	0.28	2.42*
	Police classification				−0.06	−0.08	−0.03	−4.26***
	Education level				0.03	−0.06	0.11	0.59
	POS				0.46	0.40	0.52	15.03***
	RESE				0.33	0.27	0.39	11.11***
	POS × RESE				0.07	0.03	0.12	3.00**
WE	Gender	0.70	0.50	95.06***	−0.02	−0.15	0.11	0.80
	Police classification				−0.04	−0.07	−0.01	−3.02^∗∗^
	Education level				0.11	0.02	0.20	2.47*
	JS				0.61	0.53	0.69	15.30***
	POS				−0.04	−0.11	0.03	−1.07
	RESE				0.15	0.08	0.22	4.50***
	JS × RESE				0.05	0.01	0.10	2.15*
	POS × RESE				−0.02	−0.08	0.04	−0.75

**N* = 744; WE, work engagement; POS, perceived organizational support; JS, job satisfaction; RESE, regulatory emotional self-efficacy. All the variables in the model were normalized and then substituted into the regression equation. LLCI, lower level confidence interval. ULCI, upper level confidence interval. ^∗^*P* < 0.05, ^∗∗^*P* < 0.01, ^∗∗∗^*P* < 0.001.*

**FIGURE 4:**
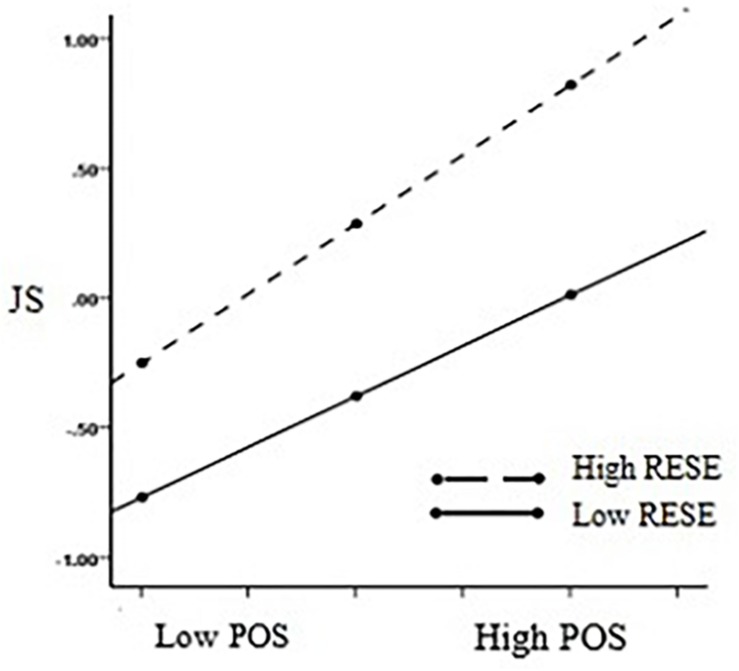
Simple slopes of Regulatory emotional self-efficacy moderate the relationship between Perceived organizational support and Job satisfaction. *N* = 744, POS, perceived organizational support; JS, job satisfaction; RESE, regulatory emotional self-efficacy.

**FIGURE 5:**
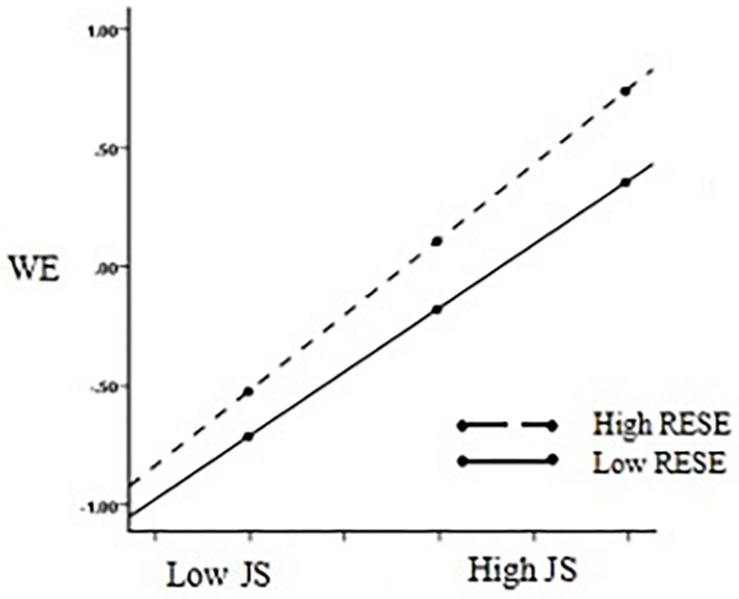
Simple slopes of Regulatory emotional self-efficacy moderate the relationship between Job satisfaction and Work engagement. *N* = 744, JS, job satisfaction; WE, work engagement; RESE, regulatory emotional self-efficacy.

Furthermore, we used a simple slope test to analyze the moderating effect of regulatory emotional self-efficacy on perceived organizational support and job satisfaction (see Figure 4), and the moderating effect of regulatory emotional self-efficacy on job satisfaction and work engagement (see Figure 5). With the increase in the level of regulatory emotional self-efficacy, organizational support’s positive prediction effect on job satisfaction gradually expanded (β = 0.39, *t* = 10.17, *p* < 0.001, 95% bootstrap CIs [0.31,0.46] →β = 0.54, *t* = 13.37, *p* < 0.001, 95% bootstrap CIs [0.45,0.61]). Hypothesis 2a was therefore supported. In addition, job satisfaction’s positive predictive effect on work engagement gradually rose along with the increase in the level of regulatory emotional self-efficacy (β = 0.54, *t* = 13.31, *p* < 0.001, 95% bootstrap CIs [0.46,0.62] →β = 0.63, *t* = 14.91, *p* < 0.001, 95% bootstrap CIs [0.55,0.72]). Hypothesis 2b was therefore supported.

The indirect effect of perceived organizational support on work engagement *via* job satisfaction was statistically significant for the police officers with low regulatory emotional self-efficacy (i.e., one standard deviation below the mean), β = 0.25, *SE* = 0.03, 95% bootstrap CIs = [0.18,0.32]. And this indirect effect was significant for police officers with high regulatory emotional self-efficacy (i.e., one standard deviation above the mean), β = 0.39, *SE* = 0.04, 95% bootstrap CIs = [0.31,0.48]. With the improvement of police’s regulatory emotional self-efficacy, the indirect effect of perceived organizational support on work engagement *via* job satisfaction is gradually enhanced.

In sum, the moderated mediation model was established (Hayes, 2012), Hypothesis 3 was supported. Job satisfaction plays a mediating role in perceived organizational support and work engagement. What’s more, regulatory emotional self-efficacy not only moderates the relationship between perceived organizational support and job satisfaction but also moderates the relationship between job satisfaction and work engagement.

## Discussion

### The Relationship Between Perceived Organizational Support and Chinese Police Officers’ Work Engagement

The results showed a significant, positive correlation between police organizational support and work engagement. This implies that police officers perceived a higher level of support from the organization. Thus, they will put more effort into their work and be more involved in it.

Previous research has also found a significant, positive correlation between perceived organizational support and work engagement; in turn, this affects the latter (Sulea et al., 2012; Zhong et al., 2015; Gillet et al., 2017). According to the JD-R model, when job resources (such as organizational support and justice) are not sufficient to meet the demands of the job, work engagement will decrease (Demerouti et al., 2001; Bakker et al., 2004). This study also agrees with the predictions from the COR theory, the company provides employees with assistance so that they can stay motivated and maintain good working conditions, thereby reducing the physical and mental consumption of resources and burnout at work, enabling them to strive and focus on their tasks.

As a special profession, police work is related to social safety and stability. Compared to other vocations, police officers face unpredictable emergencies and experience significant amounts of pressure. It is therefore important to strengthen organizational support for police officers and help them perceive that they are receiving more help from the organization and an increase in their working resources (such as organizational support). This plays a particularly important role in increasing the work engagement of police who work in such high work pressure environments.

### The Mediating Role of Job Satisfaction

The outcomes demonstrate that job satisfaction plays a mediating role in perceived organizational support and work engagement. In other words, after adding the mediating variable of job satisfaction, perceived organizational support does not directly promote work engagement, but indirectly affects it through job satisfaction.

As a valuable organizational resource, organizational support has an important positive impact on employees’ job satisfaction. Many studies have shown that job satisfaction can be affected by acknowledging achievement, work itself, and other internal aspects (Spencer, 1982), and organizational factors (such as organizational culture and the company environment). Therefore, when the organization provides assistance to police officers and recognizes their successes, their job satisfaction will improve. Furthermore, studies have revealed that job satisfaction is the basis for work engagement (Salanova et al., 2011; Rayton and Yalabik, 2014). When individuals’ socio-emotional needs are met and they are satisfied with their work, they devote themselves to work in a more active and fuller state of mind and are able to realize their work value.

In addition to organizational/social support, external elements (such as salary and leadership style) have a significant impact on individual job satisfaction. In a survey on salary conducted with 7,612 respondents from the United States, Britain, Japan, and additional countries, nearly half of the respondents said that low availability of funds reduces their job satisfaction (Smaglik et al., 2014). Moreover, many researchers believe that the daily behaviors of supervisors positively affect the tact of the working environment and stimulate employees’ work engagement in this case. For example, in Breevaart et al.’s (2014) study, it was found that after controlling for followers’ work engagement the previous day, employees were more engaged on the following days when their leader took a more transformational leadership approach and provided contingent rewards. In addition, the leader exhibiting a sense of humor created a more pleasant and fun working atmosphere with the potential to help employees generate positive emotions at work, thereby increasing their work engagement (Goswami et al., 2016).

As a high-pressure professional group, police officers’ work objectives and content are more troublesome, and it is easy for them to encounter setbacks. Additionally, in China, the police’ salary system is fixed and stable, so the emotional support of the organization is very important. According to the results of this study and the above opinions, if the organization, and particularly its leaders, can use positive management methods in the management process and give timely spiritual encouragement and material protection, tasks are more likely to be successfully completed. When the emotional needs of police officers are met, they are more satisfied with their work, and will evoke a fuller and more active spirit in their work.

### The Moderating Role of Regulatory Emotional of Self-Efficacy

This study also found that in its indirect effect (perceived organizational support on work engagement via job satisfaction), high regulatory emotional self-efficacy was stronger than that of low regulatory emotional self-efficacy. Specifically, first, when the police officers’ regulatory emotional self-efficacy was low, the positive predictive effect of organizational support on job satisfaction was weak; when the police officers’ regulatory emotional self-efficacy was high, the positive predictive effect of organizational support on job satisfaction was enhanced. Second, when the police officers’ regulatory emotional self-efficacy was low, the positive predictive effect of job satisfaction on work engagement was weak; when the police officers’ regulatory emotional self-efficacy was high, the positive predictive effect of police job satisfaction on work engagement was enhanced.

As positive psychological resources can be used by individuals, personal resources strengthen the positive role between job resources and work engagement. The findings of this study are consistent with previous outcomes regarding the protective effect of regulatory emotional self-efficacy (e.g., Bandura et al., 2003; Yuan et al., 2017). Regulatory emotional self-efficacy plays an important role in helping the police to face with negative emotions caused by inadequate organizational support and job dissatisfaction. Furthermore, studies have shown that in addition to organizational factors, elements of personality can also affect job satisfaction (Ilies and Judge, 2002). Therefore, when police officers perceive that organizational support for their work is low, but their level of regulatory emotional self-efficacy is high (vs. low regulatory emotional self-efficacy), then their ability to regulate their emotions can buffer (or reduce) the impact of organizational support on job satisfaction. In contrast, even if police officers believe that the organization provides strong support for their work, their poor emotional regulation may decrease job satisfaction.

According to Demerouti and Bakker (2011), work and personal resources are the main predictors of work engagement. Job resources (such as assistance from the organization), and personal resources (such as self-efficacy) are two predictors of work engagement. However, even though police officers may be satisfied with their work, they may not have the skills to deal with their emotions (such as having a low ability to express or handle feelings), they may be less engaged in their work and may perform poorly, possibly resulting in experiencing job burnout or resignation. On the contrary, police officers with a high level of self-efficacy in emotion regulation may put more effort into their work. The broaden-and-build theory of positive emotions can activate behavior, expand cognitive construction resources, and relieve stress, while negative emotions have the opposite effect (Fredrickson, 1998). Moreover, the broaden-and-build theory also indicated that positive emotions could eliminate the lingering influence of negative ones (Fredrickson, 2001). The ability of positive emotion expression can help alleviate dissatisfaction police may have with their work, can maintain a good state of work engagement, and can effectively help in completing work tasks. In high-stress professions such as police work, it is especially necessary to use positive emotions to reduce the impact of negative emotions.

In order to improve police officers’ work engagement, the organization can on the one hand develop psychological behavioral training according to the individual factors of the officers. A study on self-regulation behavior, self-efficacy, and psychological skills training for military practice pilots, showed that self-regulation behavior increased linearly after intervention. In addition, significant increases in self-efficacy and psychological skill use were observed, along with concomitant reductions in anxiety. After 2 months of follow-up, the change was still present (Mccrory et al., 2013). Therefore, according to the occupational characteristics of police work with high pressure, risk, and stress, it is absolutely necessary to train police officers in psychological behavioral training. On the other hand, organizations can use medium-term interventions to provide positive guidance to police officers on how to play to their strengths in their work, potentially raising both positive awareness of themselves as members of the organization and engagement in their work. In an intervention study of organization-based self-esteem and work engagement (Costantini et al., 2019), researchers develop and channel employees’ strengths by inspiring participants on how to positively reframe emotional situations, supporting them in the identification of possible challenging work situations, and structuring reflection and meaning-making processes to support their awareness of work identity beliefs. The research results show that after 9 months (three phases) of intervention, employees who feel valued and appreciated in their working contexts feel energetic, dedicated, and inspired in their work.

Based on the above findings, in addition to increasing support for police work and to improve the perception of being valued and appreciated in the working environment, the organization unit should also carry out vocational training in psychological skills for police officers. Strengthening the ability of the police to express positive emotions (such as relaxation and pleasure) is of great significance in reducing negative emotions (such as anxiety and depression) in daily work, which helps to promote individuals to be more engaged in their work. This may improve their sense of identity as members of the police organization and their job satisfaction, leading to more active involvement in their work. This is crucial to maintaining social order and stability.

## Limitations and Strengths

This study attempted to explain the relationship between organizational support (organizational resource) and work engagement by examining job satisfaction among police officers. Although our findings provide empirical support for strengthening police work engagement, this study has several limitations. First, although our study is mainly based on the JD-R model and takes into account the COR theory, the cross-sectional design adopted here is not a longitudinal experiment, thus making it highly limited, and any inference about causality is therefore limited. Although our study tried to explain the mechanism or process of police work engagement by using intermediary analysis, the causal relationship between the sense of organizational support and work engagement of police was not determined due to the limitations of the cross-sectional design. Second, the sample is not sufficiently representative, because our sample was composed of Chinese police officers, it may not be feasible to generalize the results to other employees and outside this country. China is a collectivist country. Being a police officer is not only a vocation, it is also a responsibility and honor. The job resources of police are very rich. But, in cases of racism and sexism, where individuals may be resource rich (Westman et al., 2004), we adopted the self-report method. There may therefore be a reference-group effect, whereby some people evaluate themselves not by global standards, but by the criteria of others in their country or culture. Last, in addition to emotion regulation self-efficacy, individual factors, such as psychological capital and psychological detachment, have the potential to influence police work engagement. Family work conflict also has an impact on police work engagement.

The results of this study have important theoretical and practical significance. First, most studies of police work engagement is done in the context of western culture, this study focuses on China exclusively, a typical collectivist nation in East Asia, studying the influencing factors of work engagement among Chinese police officers. This helps to understand differences in police participation in different cultures and whether there are common laws. What’s more, this study deepens the interaction process of work resources and personal resources in the JD-R model, and investigates police work engagement by combining organizational factors (perceived organizational support) and personal factors (regulatory emotional self-efficacy), so as to motivate police to be highly engaged in work. This has important practical significance for maintaining social security and stability and for maintaining the orderly operation of politics and economy. At the same time, this study also expands the application field of the JD-R model, especially in some special professions (e.g., police officers). It can be used as a reference to expand upon in other special professions.

## Practical Implications

This research is devoted to exploring the interactions and relationships between organizational factors and personal factors. The current research provides a certain enlightenment for improving the work engagement of police and other types of employees.

In a prison police training work, we communicated with the Chief Executive on the model built in this study, which has been recognized by them. We all agree that as a high-pressure professional group, the police’s work objectives and contents are more troublesome, and setbacks can more easily be encountered. At this time, if the organization, especially the leadership can give timely spiritual encouragement and material protection, tasks are more likely to be successfully completed. In particular, compared with the fixed and stable salary system, the emotional support of the police organization is more important, which specifically can be achieved in practice by often asking about their physical condition, accompanying them when they work overtime, discussing solutions together, so that they can really feel organizational support to meet their social and emotional needs, which will lead to them feeling satisfied with their work, and so they can eventually devote themselves to work wholeheartedly.

At the same time, we should also see that the support provided by the organization is the same, but because of the different emotional states of the individual police, this support may also be discounted. Therefore, the training of emotional regulation ability is particularly necessary in police training. In a training session, we taught police some skills of emotional regulation, such as cognitive adjustment, music appreciation, positive self-suggestion and so on.

The general pattern found in the present study suggests that job resources and personal resources are particularly important to motivate police. Because previous research has suggested that employees who are actively involved rarely withdraw from the organization, and have a lower sense of burnout (Schaufeli and Bakker, 2004), it can be concluded that police organizations and personnel may benefit from investment in the resources at work, and the integration of work resources and personal resources.

## Conclusion

This study establishes a mediating model of regulation from organizational and individual components to explore police work engagement. Model analysis explains how perceived organizational support affects police work engagement. Furthermore, it illustrates which individuals are more aware of how perceived organizational support impacts police work engagement through job satisfaction. This improves the model’s explanatory power.

Based on our analysis and discussion, we suggest that job satisfaction plays a mediating role between perceived organizational support and work engagement. Regulatory emotional self-efficacy moderated the first and later halves of the mediation process. Our results provide both a theoretical and empirical basis for the development of interventions, first, by strengthening organizational support for police and helping them perceive that they are receiving more company assistance and second, the leaders of police organizations should use positive management methods in the management process to satisfy the emotional needs of police. Last, police need to strengthen their ability to express positive emotions and to boost their capacity to manage negative ones. This is vital in improving police work engagement.

## Data Availability Statement

The raw data supporting the conclusions of this article will be made available by the authors, without undue reservation, to any qualified researcher.

## Ethics Statement

The studies involving human participants were reviewed and approved by Ethics Committee of the College of Psychology at Jiangxi Normal University. The patients/participants provided their written informed consent to participate in this study.

## Author Contributions

TLa and XZ contributed to the conception and design of the study. TLa and MC performed the statistical analysis. TLa wrote the first draft of the manuscript. XZ and MC revised it critically for important intellectual content. TLa and TLi collected the raw data and organized the database.

## Conflict of Interest

The authors declare that the research was conducted in the absence of any commercial or financial relationships that could be construed as a potential conflict of interest.
